# COVID-19 Help-Seeking Behaviors: Application of the Theory of Planned Behavior

**DOI:** 10.5334/pb.1034

**Published:** 2021-12-29

**Authors:** Mohammed Aldalaykeh, Mohammed M. Al-Hammouri, Jehad Rababah, Tariq Al-Dwaikat

**Affiliations:** 1 Jordan University of Science and Technology, Community & mental Health nursing Department, JO; 2 Jordan University of Science and Technology, Adult health nursing Department, JO

**Keywords:** COVID-19, Help-seeking, Theory of Planned Behavior, Intention, Knowledge

## Abstract

The novel coronavirus disease (COVID-19) is highly contagious. More than 247 million cases have been confirmed by the end of October 2021. Seeking help earlier may slow the spread of COVID-19 because it may help in early detection of infected cases, and it facilitate tracing those who were with close contact with infected cases. The purpose of this study is to identify participants’ intentions toward COVID-19 seeking help and the factors affecting their decision. This is a cross-sectional study. An online survey using Google Forms was used for data collection. Hierarchical multiple regression analysis was used to explain intentions to seek help for COVID-19. The concepts included in the Theory of Planned Behavior and COVID-19 knowledge were used as predictors. The sample included 780 participants, with an average age of 28.60±9.86 years old. Most of the sample were female (67.4%) and having a bachelor’s degree (72.7%). Participants showed high level of knowledge regarding COVID-19, 73% of the sample had a total knowledge score equal to or higher than 85%. Also, participants had high positive attitudes and high intentions to seek help for COVID-19. The four predictors: Attitudes towards COVID-19, subjective norm, perceived behavioral control, and COVID-19 knowledge significantly explained intentions to seek help. Participants had high intentions to seek help for COVID-19, which was related to having positive attitudes toward seeking help, high social approval, high perceived controllability, and high COVID-19 knowledge levels. Regular awareness campaigns during early stages of pandemics should be performed to improve attitudes and knowledge level, which may improve prevention measures, and promote help seeking behaviors. Consequently, this may facilitate early detection of cases, and slow the spread of pandemics.

## Introduction

The outbreak of the novel coronavirus disease (COVID-19) is now considered pandemic with total number of confirmed cases exceeded 247 million cases by the end of October 2021, and number of deaths reaching approximately 5 million (World Health Organization [WHO], 2021). COVID-19 caused negative consequences globally on individuals, communities and governments (WHO, 2020). For example, the lockdown and curfew used by most countries to slow down the spread of this virus has negative financial consequences, since most shops and factories were forced to close. Also, the psychological impact of this pandemic was strong and included severe levels of anxiety, depression, and phobia (WHO, 2020).

Jordan is a middle eastern country with a population of approximately 10.5 million (Department of Statistics, 2020). The number of COVID-19 cases in Jordan reached to approximately 862,000 cases by the end of October 2021, while the total number of deaths was 11,000 (WHO, 2021). Early and strict governmental orders were applied during the mid of March 2020 which included lockdown and complete curfew, followed by fines and risk of being imprisoned for any Jordanian who break these orders (The Jordan Times, 2020). As Jordan was approaching the end of March, the order of curfew was modified, allowing people to go out for shopping for necessary groceries between 10AM until 6PM. Afterwards, restrictions were removed gradually, and people were allowed to stay outside until 11PM. Meanwhile, preventive measures were highly recommended such as masks and social distancing (The Jordan Times, 2020). During the period December 2020 – January 2021, several COVID-19 vaccines have been approved, and were being distributed and administered globally to control the pandemic (WHO, 2021). Afterwards, Jordanians were being encouraged by the government and health officials to take the vaccine, and by the end of June 2021, the curfew order was canceled (Ministry of Health [MOH], 2021). As of November 2021, more than 3,680,000 Jordanians took the two doses of COVID-19 Vaccine (MOH, 2021).

COVID-19 is highly transmissible and contagious (Centers for Disease Control and Prevention [CDC], 2020). So, it is important to determine individuals’ intentions towards COVID-19 seeking help, because this may help healthcare professionals to act and intervene on cases at earlier stages. Help-seeking behaviors in the context of COVID-19 and in this study focuses mainly on COVID-19 PCR testing, contacting the primary care provider, and accessing health clinics or emergency departments for having symptoms similar to COVID-19 (Ravert et al., 2021; Shahrour et al., 2021). Seeking help for COVID-19 (e.g. PCR testing) as soon as the symptoms appear helps healthcare professionals to trace those who were with close contact with an infected person, and screen them and apply preventive measures for them such as quarantine and masks if their test results were negative, or start isolation and treatment if they were found to have positive results. Consequently, seeking help for COVID-19 at earlier stage of developing signs and symptoms helps in slowing the spread of this pandemic. To that effect, however, it is important to determine the factors that may affect the intention to seek help for COVID-19. Identifying these factors may provide healthcare leaders with recommendations and suggestions on how to encourage people to seek help and how to control barriers. Examples of the barriers identified in the literature included negative attitudes toward COVID-19 preventive measures and testing, discrimination or stigma if diagnosed with COVID-19, and poor knowledge regarding COVID-19 (Das et al., 2021; Noorrizki et al., 2021; Shahrour et al., 2021).

The Theory of Planned Behavior (TPB) was used to guide this study and examine participants’ intention towards seeking help for COVID-19. According to this theory, the actual behavior is highly affected by a person’s intention to perform that behavior (Fishbein & Ajzen, 2010). This theory assumes that individuals’ intentions to perform a certain behavior are influenced by: a) their attitude toward that behavior, b) subjective norm which means the social approval or disapproval of performing that behavior, and c) perceived behavioral control which means the perceived barriers and the amount of control individuals have over their decision (Fishbein & Ajzen, 2010). The TPB is one of the most commonly used theories in predicting behaviors, and it successfully predicted a wide variety of healthy behaviors in previous studies (Aldalaykeh et al., 2019; Fishbein & Ajzen, 2010; Mak & Davis, 2014; Mo & Mak, 2009; Woods, 2013). In the context of COVID-19, several studies used the TPB to predict successfully intentions and actual behaviors related to social distancing, PCR testing, and compliance with health protocols (Das et al., 2021; Gibson et al., 2021; Noorrizki et al., 2021). This theory was chosen to guide this study because it consistently showed high predictive power (i.e., explained variance), and it includes cognitive as well as social factors to predict intentions and behaviors (Aldalaykeh et al., 2019; Fishbein & Ajzen, 2010; Mak & Davis, 2014; Woods, 2013).

Previous studies showed the importance of health-related knowledge in improving help seeking behaviors (Adela et al., 2020; Al Ali et al. 2017; Batterham et al., 2013; Chan et al., 2014; Paul et al., 2020; Prasetyo et al., 2020). People who have a higher level of knowledge about a certain disease or health issue are more likely to seek help than others (Adela et al., 2020; Chan et al., 2014; Khan et al., 2020; Paul et al., 2020; Prasetyo et al., 2020). In the context of COVID-19 several studies indicated the importance of COVID-19 knowledge in improving help-seeking behaviors such as PCR testing or contacting the primary care provider and applying preventive measures such as masks, social distancing, and hand washing (Bonner et al., 2020; Boot et al., 2021; Hussein et al., 2021; McCaffery et al., 2020; Noorrizki et al., 2021; Shahrour et al., 2021) So, it is important to examine the effect of COVID-19 knowledge level on individuals’ intentions to seek help for COVID-19. Our hypothesis would be participants’ attitude toward COVID-19 seeking help, subjective norm, perceived behavioral control, and COVID-19 knowledge levels significantly explain participants’ intention toward COVID-19 seeking help. The purpose of this study is to identify participants’ intention toward COVID-19 seeking help and the factors affecting their decision.

## Methods

A cross sectional correlational design was used in this study. An online survey using Google Forms was used for data collection. The sample included Jordanians who were 18 years or older, residing in Jordan during COVID-19 outbreak. Those who were already treated for or diagnosed with COVID-19 were excluded. The online survey was posted on social media websites and applications, mainly Facebook and WhatsApp. Snowball sampling technique was used to ensure continuous, quick, and wide spread of the survey. The use of convenience sampling method in this study was more appropriate because of the difficulty to have a well-defined population, and thus create a very long list for potential participants that enable random sampling method. In other words, convenience sampling method was more feasible, especially considering restrictions during the pandemic such as curfew. Data collection started on April 11, 2020 and completed on April 20, 2020. The sample included 780 Jordanians. The sample size was calculated using G*Power software, which takes into consideration effect size, power, precision level, and number of predictors (Faul, Erdfelder, Buchner, & Lang, 2009).

Institutional Review Board (IRB) approval was obtained from Jordan University of Science and Technology. Potential participants were informed in the online survey that their participation is voluntary and no identifying information will be obtained, and information will be confidential and no access to information except for the research team. Also, they were informed that the survey is very short, and it will take no longer than 5–10 minutes.

### Participants

The sample consisted of 780 participants with an average age of 28.60±9.86 years old, participants age ranged between 18–69 years old. Most participants were female (N = 526, 67.4%). More than half of the sample were single (N = 426, 54.7%), and the majority had a bachelor’s degree (N = 566, 72.7%). Only 61 (7.8%) participants reported having chronic illnesses (***Table 1***). No significant association was found between demographic variables and participants’ intention towards COVID-19 seeking help, nor it was found with COVID-19 knowledge. Also, ***Table 1*** shows that small effect size has been achieved after analyzing the association between each demographic variable and intention towards COVID-19 seeking help.

**Table 1:** Demographic Characteristics (N = 780).


VARIABLE	CATEGORY	FREQUENCY (%)	EFFECT ON INTENTION

Gender	Female	526 (67.4)	(t = –1.66, p = 0.107, *d* = 0.165)

	Male	254 (32.6)	

Marital status	Single	426 (54.7)	(F = 1.11, p = 0.351, η^2^ = 0.013)

	Married	333 (42.6)	

	Divorced	16 (2.1)	

	Widowed	5 (0.6)	

Educational level	Primary/elementary	9 (1.0)	(F = 1.78, p = 0.294, η^2^ = 0.020)

	High school	57 (7.3)	

	Bachelor’s	566 (72.7)	

	Master’s/PhD	148 (19.0)	

Chronic illness	No	719 (92.2)	(t = –1.86, p = 0.067, *d* = 0.190)

	Yes	61 (7.8)	


### Measures

The theory of planned behavior questionnaire developed by Aldalaykeh et al., (2019) was used and adapted to the targeted behavior (COVID-19 help seeking). This survey is already in Arabic and has been used on a Jordanian sample previously. This survey includes four concepts:

1. *Attitude Towards COVID19 help seeking (ATT-COVID19)*. This scale includes 5 semantic differential items, each item has two opposite adjectives. Participants have the option to choose between 6 points and check the point that is closer to their feeling or appraisal toward COVID-19 help seeking. An example of the items used is “for me, COVID-19 help seeking (e.g., PCR testing or calling the primary care provider) is considered Very good/Very Bad”. The total score of this scale ranges between 6–30, with higher scores indicating more positive attitude towards COVID-19 help seeking. This scale showed good internal consistency (Cronbach’s alpha = 0.82) (Aldalaykeh et al., 2019). In this study, ATT-COVID19 showed also good internal consistency (Cronbach’s alpha = 0.80).
2. *Subjective Norm (SN)*. This scale measures the social influence of family, friends, and relatives on participant’s decision to seek help for COVID-19. SN included three items; each item has 6-point Likert scale. An example of the items used is “Most people who are important to me think that I should seek help for COVID-19 (e.g., PCR testing or calling the primary care provider) if I show typical signs and symptoms”. The total score ranges between 3–18, with higher scores indicating higher social approval for COVID-19 help seeking. This scale approached acceptable levels of internal consistency in a previous study (Cronbach’s alpha = 0.69) (Aldalaykeh et al., 2019). In this study, SN showed acceptable internal consistency (Cronbach’s alpha = 0.74).
3. *Perceived Behavioral Control (PBC)*. This scale measures the barriers of COVID-19 help seeking and the level of control a person has over his/her decision to seek help for COVID-19 independently and voluntarily. PBC included three items, each item has 6-point Likert scale. An example of the items used is “The decision to seek help for COVID-19 depends solely on me”. The total score ranges between 3–18, with higher scores indicating higher ability and controllability to seek COVID-19 screening test. PBC showed acceptable level of internal consistency (Cronbach’s alpha = 0.71) (Aldalaykeh et al., 2019). In this study, PBC showed acceptable internal consistency (Cronbach’s alpha = 0.70).
4. *COVID-19 help seeking intention (COVID-19 IN)*. This scale measures people’s intention towards COVID-19 help seeking. COVID-19 IN included three items, each item has 6-point Likert scale. An example of the items used is “I plan to seek help for COVID-19 (e.g., PCR testing or calling the primary care provider) if I develop typical signs and symptoms”. The total score ranges between 3–18, with higher scores indicating higher intention towards COVID-19 help seeking. Acceptable levels of internal consistency were reported previously for this scale (Cronbach’s alpha = 0.76) (Aldalaykeh et al., 2019). In this study, COVID-19 IN showed acceptable internal consistency (Cronbach’s alpha = 0.78).
5. *COVID19 knowledge*. As no existing tools were found at the time of conducting this study that measured COVID-19 knowledge, a tool was developed by the researchers to measure participants’ level of knowledge regarding the signs and symptoms of COVID-19. This tool was developed to estimate the ability of the participant to differentiate between the correct signs/symptoms of COVID-19 and unrelated signs/symptoms. According to WHO (2020) the three most common signs/symptoms of COVID-19 are fever, dry cough, and shortness of breath (dyspnea). The tool included these three signs/symptoms along with four unrelated symptoms (sneezing, itchy skin, red eyes, and vomiting). It included a total of seven signs/symptoms, and participants were asked to select only the correct signs/symptoms that are associated with COVID-19. There are 3 correct choices and 4 false choices. The total score is calculated by summing correct answers (range = 0–7) and transforming it to a percentage. So, the range of scores is between 0–100% with higher scores indicating higher knowledge about COVID-19 signs and symptoms. This tool has been reviewed by a panel of Jordanian experts in terms of face and content validity.

### Data analysis

Statistical Package for the Social Sciences Software (SPSS, version 23.0) was used for data analysis. Descriptive statistics were used to identify demographic characteristics of the sample, and to calculate the frequency, mean scores, and ranges of the concepts used in this study. Several tests such as t-test, ANOVA, and Pearson correlation were used to examine the effect of demographic variables on the dependent variable (COVID-19 help seeking intention). Bivariate correlation analysis was used to create a correlation matrix using Pearson Correlation. Hierarchical multiple regression analysis was used to predict the effect of the TPB concepts and COVID19 Knowledge on COVID-19 help seeking intentions. The first step included three predictors: ATT-COVID19, SN, and PBC, while the second step examined the effect of adding COVID19 Knowledge as a predictor (4 predictors in the second step). Assumption of multicollinearity was tested using tolerance and VIF, and they were not violated.

## Results

**Figure 1:**
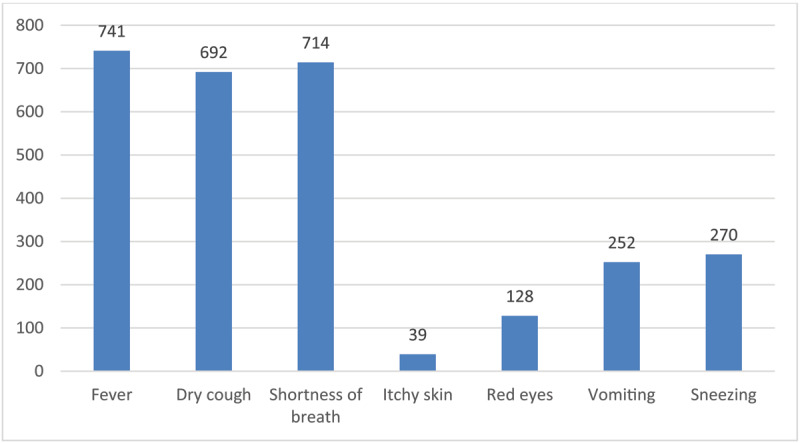
Frequency of correct signs/symptoms of COVID-19.

***Figure 1*** shows the frequency of correct signs/symptoms of COVID-19 as reported by the participants. Participants showed high knowledge level regarding COVID-19 signs/symptoms, as the most common and true symptoms were endorsed by the majority of participants. Fever, dry cough, and shortness of breath (dyspnea) were endorsed by 95.1%, 88.8%, and 91.7% of participants respectively (***Figure 1***). Also, the participants were able to differentiate that sneezing, itchy skin, red eyes, and vomiting are not related to COVID-19. For example, only 5% and 16.4% of participants reported itchy skin and red eyes respectively as correct signs/symptoms of COVID-19 (***Figure 1***). The mean total score of COVID19 Knowledge was 85.47%±14.20, and it ranged between 42.86%-100% (***Table 2***). Approximately, 73% of the sample have a total knowledge score equal to or higher than 85%.

**Table 2:** Description of study variables (N = 780).


VARIABLE	MEAN	SD	RANGE	MEDIAN

ATT-COVID19	26.85	3.45	5–30	28.00

SN	15.07	2.82	3–18	16.00

PBC	13.35	3.62	3–18	13.00

COVID19 Knowledge	85.47	14.20	42.86–100	85.70

COVID-19 IN	15.69	2.76	3–18	16.00


ATT-COVID19: Attitude Towards COVID19 help-seeking behavior. SN: Subjective Norm. PBC: Perceived behavioral control. COVID-19 IN: COVID-19 help-seeking intention.

Participants had high positive attitude towards seeking help for COVID-19 (***Table 2***). Also, they reported a high social approval (SN) from family and significant others toward seeking help. In addition, they reported a high ability (PBC) to seek help if they develop the typical signs and symptoms, and high intentions towards COVID-19 help-seeking.

### Regression analysis

The first step to run a regression analysis is to measure the association between the major concepts of the study. Bivariate correlation between the TPB concepts and COVID19 Knowledge are shown in ***Table 3***. COVID-19 help-seeking intention was significantly and positively associated with all other concepts. COVID-19 help seeking intention was strongly correlated with ATT-COVID19 (r = 0.499), and moderately correlated with both SN (r = 0.414) and PBC (r = 0.321). COVID-19 help-seeking intention was associated with COVID19 Knowledge, but it showed small effect (r = 0.176).

**Table 3:** Correlation matrix of the major concepts in the study (N = 780).


CONCEPTS	ATT-COVID19	SN	PBC	COVID19 KNOWLEDGE

SN	0.401***			

PBC	0.249***	0.076*		

COVID19 Knowledge	0.135*	0.039	–0.011	

COVID-19 IN	0.499***	0.414***	0.321***	0.176**


* < 0.05 ** < 0.01 *** < 0.001. ATT-COVID19: Attitude Towards COVID19 screening test. SN: Subjective Norm. PBC: Perceived behavioral control. COVID-19 IN: COVID-19 help-seeking intention.

***Table 4*** shows the result of hierarchical regression analysis to explain the participants’ intention towards COVID-19 help-seeking. The assumption of multicollinearity was not violated since tolerance scores were higher than 0.10, and VIF scores were lower than 10. The first step included three predictors: ATT-COVID19, SN, and PBC, and they represent the original model of the TPB. The first step significantly predicted intentions toward seeking help (F = 60.788, p < 0.001). The first step accounted for 34.0% of explained variance (Adjusted R^2^ = 0.340), and it showed a large effect size (ƒ^2^ = 0.520). All variables included in the first model showed significant independent prediction effect (***Table 4***). The largest effect was related to participants’ attitude toward COVID-19 seeking help, followed by subjective norm, and then perceived behavioral control (***Table 4***). The second step included four predictors by adding COVID19 Knowledge. The second step significantly predicted COVID-19 intentions toward seeking help (F = 48.566, p < 0.001), and the four predictors accounted for 35.3% of explained variance (Adjusted R^2^ = 0.353). The second step also significantly improved prediction of intentions (F change = 8.130, p = 0.005), and it showed a large effect size (ƒ^2^ = 0.546). All four predictors in the second model showed significant independent effect on intentions. ATT-COVID19 was the strongest predictor, followed by SN and then PBC.

**Table 4:** Hierarchical regression analysis of COVID-19 help seeking intentions (N = 780).


MODEL	ß	ADJUSTED R^2^	F	F CHANGE

1 ATT-COVID19	0.343***	0.340	60.79***	60.79***

SN	0.257***			

PBC	0.212***			

2 ATT-COVID19	0.324***	0.353	48.57***	8.13**

SN	0.259***			

PBC	0.218***			

COVID19 Knowledge	0.124**			


* < 0.05 ** < 0.01 *** < 0.001. ATT-COVID19: Attitude Towards COVID19 screening test. SN: Subjective Norm. PBC: Perceived behavioral control. COVID-19 IN: COVID-19 help-seeking intention.

## Discussion

This study used the concepts of the TPB along with COVID-19 knowledge level to explain intentions to seek help. The results indicated that participants had high and strong intentions towards seeking help for COVID-19 including PCR testing, contacting the primary care provider, and accessing healthcare clinics or emergency departments. This is consistent with the findings of several other studies in other countries (Campbell et al, 2021; Ravert et al., 2021; Shahrour et al., 2021). This could be related to several reasons. First, the findings indicated that participants had very high positive attitude towards help-seeking. Second, they showed high social approval from family and significant others towards help-seeking. The Jordanian culture is collectivist in nature, and this may have increased the effect of subjective norm on intentions (Aldalaykeh et al., 2019; Al Ali et al., 2017). Third, participants showed a high control over their decision to screen themselves and they also reported lower barriers toward seeking help. This could be related to lower cost of treatment, as 72% of Jordanians have health insurance (DOS, 2020; MOH, 2021). For example, the PCR is test is free for suspected cases and the cost of healthcare services is affordable even if the person does not have health insurance (DOS, 2020; MOH, 2021). Fourth, the government and non-profit organizations conducted several campaigns through local TV channels and social media that explained all details about COVID-19 such as signs/symptoms, methods of transmission, prevention methods, and treatment approaches (The Jordan Times, 2020). These campaigns could improve the knowledge level about this pandemic, and this was supported by the high knowledge level the participants had about COVID-19 signs and symptoms.

The results of this study showed a significant and positive association between COVID-19 knowledge and the intention to seek help. This is consistent with previous studies that showed a positive association between high knowledge levels and help-seeking intentions or behaviors (Adela et al., 2020; Al Ali et al. 2017; Batterham et al., 2013; Chan et al., 2014; Paul et al., 2020; Prasetyo et al., 2020). In the context of COVID-19, researchers found that the intentions or actual behaviors of PCR testing, social distancing, and complying with COVID-19 health protocols are highly affected by the level of COVID-19 knowledge, whereas poor COVID-19 knowledge is considered a barrier in adopting these healthy and preventive measures (Bonner et al., 2020; Boot et al., 2021; Hussein et al., 2021; McCaffery et al., 2020; Noorrizki et al., 2021; Shahrour et al., 2021).

The findings of the study showed that participants have a high knowledge level about COVID-19. This is consistent with previous studies that showed a high knowledge level regarding COVID-19 in many countries including Egypt, Indonesia, USA, Cameron, Pakistan, Philippines, and some European countries (Adela et al., 2020; Boot et al., 2021; Campbell et al., 2021; Hussein et al., 2021; Khan et al., 2020; Prasetyo et al., 2020; Sari et al., 2021). The high knowledge levels in this sample could be explained by sample characteristics since the majority were relatively young and having a bachelor’s degree. This may facilitate understanding of the information related to COVID-19 and the easy access to this information through the use of social media. Also, the timing of the data collection (mid of April 2020) allowed participants to listen and watch awareness raising campaigns about COVID-19 regularly since the beginning of the pandemic, which may have enhanced their retention of information and improved their knowledge levels. Finally, high knowledge level could be related to the unidimensional measurement approach used to estimate knowledge level. In this study, we measured knowledge level by examining only COVID-19 signs and symptoms. If we measured other dimensions such as preventive measures (masks, hygiene, social distancing, etc.), treatment approaches, or complications, knowledge levels may have been more variable.

The findings of this study supported the effectiveness of the theory of planned behavior in explaining intentions to perform healthy behaviors, as evidenced by the large explained variance of the dependent variable. This is consistent with previous studies that showed significant results of this theory in explaining intentions and wide variety of behaviors (Aldalaykeh et al., 2019; Fishbein & Ajzen, 2010; Mak & Davis, 2014; Mo & Mak, 2009; Şimekoğlu & Lajunen, 2008; Thornton & Calam, 2010; Woods, 2013). In the context of COVID-19, our results were consistent with other studies that used the TPB to successfully explain intentions toward PCR testing, social distancing, and complying with COVID-19 health protocols (Das et al., 2021; Gibson et al., 2021; Noorrizki et al., 2021). Another strength of the TPB is that its original variables showed high explained variance in the first step of regression model, and although the extension of the model by adding COVID-19 knowledge improved the explained variance, but it was modest. The TPB may help healthcare leaders and policymakers to focus their efforts on improving attitudes (e.g. through awareness campaigns) and decreasing the barriers to healthy behaviors or preventive measures during this pandemic or any future pandemics to ensure higher intentions and ultimately higher achievement of the actual behaviors (i.e. decrease the gap between intentions and behaviors).

Limitations of this study include the use of a convenience sampling method and online survey, and thus, the generalizability of the findings is limited only to those who have similar characteristics. Another limitation is the use of a cross-sectional design, which does not support causality. However, one of the strengths of this study is that all tested hypotheses are based on a well-established theory, which may help establish some bases of causality, especially with the use of multiple regression. One of the limitations is the use of unidimensional tool to assess COVID-19 knowledge level. However, no readily available tool was found during the time of the study. Another limitation is measuring intentions rather than the actual seeking behavior, although Ajzen clarified that that there is a strong positive correlation between intentions and actual behavior, many studies showed that there is a gap between the two concepts (Fishbein & Ajzen, 2010). Future studies should consider the use of longitudinal designs, random sampling, and a multi-dimensional tool to assess the COVID-19 knowledge level, and measure the actual behavior.

## Conclusion

Participants showed a high level of knowledge regarding the COVID-19 signs and symptoms. This may positively affect their attitudes toward seeking help, leading to high intentions toward COVID-19 help-seeking. Also, high intentions were related to the strong social approval toward seeking help, and high perceived ability and control over the decision to seek help. To decrease the gap between intentions and actual behavior, regular awareness raising campaigns during early stages of the pandemic should be performed to improve knowledge level about the pandemic which may improve prevention measures, and promote help seeking behaviors. Consequently, this may facilitate early detection of cases, and slow the spread of COVID-19.
